# Four decades of hydroclimate-driven change and ecological condition in a Baltic raised bog assessed with Landsat and RSEI

**DOI:** 10.1038/s41598-026-44890-x

**Published:** 2026-03-25

**Authors:** Cichosz Radosław, Łyszczarz Stanisław, Jasik Michał, Szymański Norbert, Małek Stanisław

**Affiliations:** Department of Ecology and Silviculture, University of Agriculture in Kraków, Al. 29 Listopada 46, Kraków, 31-425 Poland

**Keywords:** Wetlands, Remote sensing, Hydrological conditions, Bog degradation, Climate sciences, Ecology, Ecology, Environmental sciences, Hydrology

## Abstract

Peatlands are key carbon stores but are highly vulnerable to hydroclimatic change and drainage legacies. We combined TerraClimate water-balance variables with Landsat Collection 2 surface reflectance imagery to quantify four decades (1984–2024) of land-cover change and ecological condition in the Bagna Izbickie Baltic raised bog (northern Poland). Land cover was mapped in Google Earth Engine using a Random Forest classifier into forest, bog and grassland classes, and ecological condition was assessed with the Remote Sensing Ecological Index integrating greenness, wetness, heat and dryness components. Hydroclimate showed progressive summer warming and an increasing atmospheric water demand, while precipitation remained strongly variable. The landscape reconfigured markedly: forest expanded from 52.4% (461.8 ha) in 1984 to 83.8% (738.4 ha) in 2024, whereas bog declined from 37.2% (327.9 ha) to 10.2% (89.8 ha). Despite this contraction, the reserve-level ecological index increased significantly over time, reflecting widespread greening and reduced surface exposure. However, bog condition was strongly climate-sensitive, with moisture signals remaining unstable and responses to warm/dry summers contrasting with those of forest and grassland. The results highlight that apparent greening can mask hydrological vulnerability and underscore the need for continued rewetting and control of woody encroachment.

**Supplementary Information:**

The online version contains supplementary material available at 10.1038/s41598-026-44890-x.

## Introduction

The growing awareness of the critical role peatlands play within terrestrial ecosystems, particularly in atmospheric carbon dioxide (CO₂) sequestration and in the conservation of plant and animal species characteristic of wetland habitats, has intensified the search for improved methods of ecological monitoring of peatlands and other wetland areas. Although peatlands cover approximately 3% of the Earth’s land surface^1^, they possess a disproportionately high capacity for storing soil organic carbon compared to other ecosystems^2^. Their ability to sequester CO₂ and to store it almost indefinitely in the form of peat is of paramount importance in the context of climate change mitigation and reduction of atmospheric greenhouse gases^3^. While peatlands offer significant potential in moderating climate change, they are themselves highly sensitive to its impacts. Ombrotrophic bogs - peatlands located in watershed areas and reliant exclusively on precipitation for their hydrological inputs - are particularly vulnerable to climate-induced alterations^4^. In the context of increasing risks associated with prolonged and severe drought events in temperate zones^5,6^, the water balance of peatlands demands special attention. Effective conservation and restoration of wetlands, along with the development of reliable environmental monitoring tools, are critical for safeguarding these ecologically valuable habitats. Traditional wetland monitoring approaches rely on in-situ field surveys, which provide detailed insights into habitat conditions^7^. However, such methods are limited in applicability across large, remote, and often inaccessible landscapes and are typically resource-intensive^8,9^. Consequently, remote sensing (RS) techniques are being rapidly advanced for the monitoring, mapping, and classification of wetland habitats. These approaches utilize various airborne and satellite-based datasets, including multispectral and hyperspectral imagery, synthetic aperture radar (SAR), and Light Detection and Ranging (LiDAR) data^10,11^. Compared to field-based methods, RS offers a more efficient, scalable, and cost-effective means of acquiring habitat information. Nevertheless, wetland habitats pose unique challenges for remote sensing. High internal heterogeneity within individual wetland types can result in pixel-level misclassification, while irregular and diffuse habitat boundaries complicate delineation^12^. Furthermore, multispectral satellite-based monitoring and classification of wetlands and peatlands are constrained by temporal variability in surface reflectance, driven by frequent fluctuations in moisture regimes and vegetation condition^13^.

This study focuses on the classification of a raised bog ecosystem using Landsat satellite imagery and assesses its ecological dynamics over time by employing the Remote Sensing Ecological Index (RSEI) developed by Xu (2013)^14^. This study may give a new insight on the usefulness of comprehensive satellite-based indices for assessing changes at the scale of very small objects. In addition, it demonstrates changes in the condition of peatlands and associated habitats (swamp forests) following the cessation of extraction activities and the implementation of active and passive conservation measures. The Bagna Izbickie Reserve is a typical example of the Natura 2000 habitat 7120 “Degraded raised bogs capable of natural and assisted regeneration”, which has been degraded as a result of human activity and was subsequently placed under protection. For this reason, it constitutes an ideal site for the long-term assessment of conservation measures.

Here, we combine a multi-decadal Landsat time series with drought and water-balance variables from TerraClimate to quantify long-term land-cover change and ecological condition in a raised bog landscape. We hypothesize that increasing hydroclimatic water deficit is associated with peatland drying and a shift toward woody vegetation, reducing the extent of open peatland habitats. We further hypothesize that the Remote Sensing Ecological Index (RSEI) captures interannual variability in surface ecological condition, but that its interpretation for peatland status requires explicit stratification by land-cover class because vegetation densification may increase RSEI even as peatland character is lost. Together, these analyses provide a framework for linking long-term wetland change to hydroclimate while evaluating the strengths and limits of RSEI for peatland monitoring.

## Materials and methods

### Study site and historical background

**Fig. 1:**
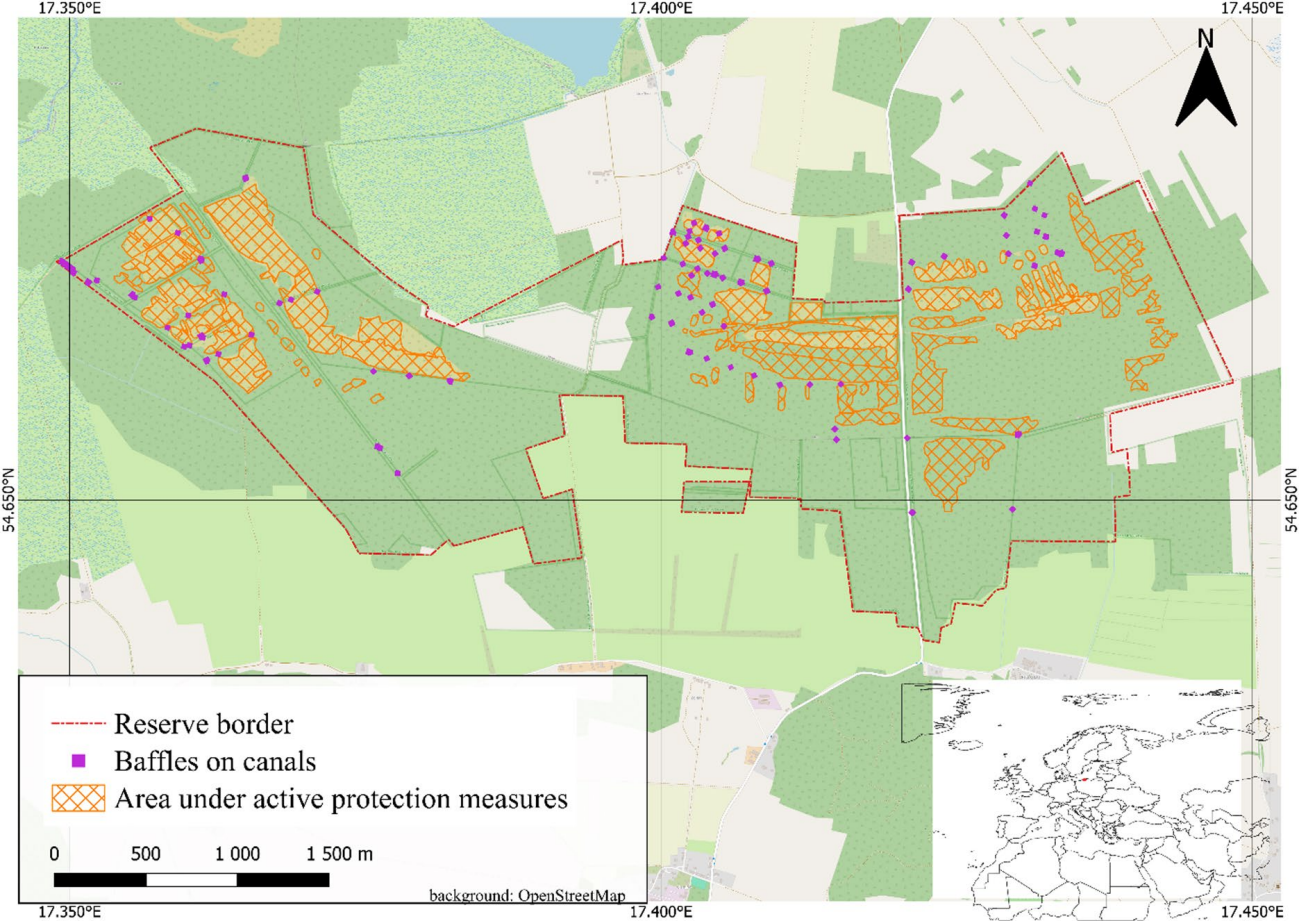
Area of the Bagna Izbickie Reserve under active protection and baffle placement. Map generated using QGIS ver. 3.34.3 (qgis.org) based on data provided by Regional Directorate of Environmental protection in Gdańsk.

This study was conducted in a Baltic raised bog located in northern Poland, within the Bagna Izbickie Nature Reserve (54°39’28.1"N, 17°25’13.4"E), situated on the Łebsko-Gardno Lowland, south of Lake Łebsko. The study site represents a typical ombrotrophic peatland of the Baltic raised bog type. The peat bog is represented by habitats belonging to the order *Oxycocco-Sphagnatea*, while the forests occurring there comprise the plant communities *Vaccinio uliginosi–Pinetum* and *Vaccinio uliginosi–Betuletum pubescentis*. Stratigraphic investigations indicate that peat formation at this site began during the Boreal period, approximately 8,000–9,000 years ago^15^. During this early stage, the peat-forming area was substantially larger, as evidenced by peat layers found in the lakebed sediments of Lake Łebsko, radiocarbon-dated to around 9,700 BP ^16^. The shifting extent of peatlands across the Łebsko-Gardno Lowland is associated with sea level fluctuations in the Baltic Sea during its transgressive phases between 7,500 and 5,000 BP ^17^. The formation of the Bagna Izbickie peatland resulted from direct paludification, a process involving waterlogging on poorly permeable mineral substrates under conditions of high annual precipitation (> 700 mm)^18,19^. The presence of compact, glacial till soils in the region has provided ideal conditions for such water retention and subsequent peat accumulation^20^. The stratigraphy of Bagna Izbickie follows the classic sequence of Baltic-type raised bogs: basal layers consist of organic gyttja, overlain by minerotrophic sedge and reed fen peat, transitioning to mesotrophic moss-sedge peats, and culminating in ombrotrophic *Sphagnum*-dominated raised bog peat. Peat thickness reaches up to 8.4 m, with a mean depth of approximately 2.5 m^19^. Hydrological alterations in the Łebsko-Gardno Lowland began in the late 18th century with the introduction of drainage networks aimed at dewatering the bogs and diverting water into Lake Łebsko. These interventions were primarily intended to facilitate peat extraction and increase the availability of pasturelands. However, until the early 20th century, such changes had minimal impact on the peatland hydrology and spatial extent. A marked transformation occurred in the first half of the 20th century, driven by agricultural intensification and the expansion of rural settlements. This necessitated more aggressive drainage efforts to convert wetland areas into arable land and to implement irrigation infrastructure. At that time, the density of drainage ditches increased to over 20 km/km², and most natural rivers were modified. Additionally, 21 polders equipped with pumping stations were constructed to provide flood control by transferring excess water from precipitation and snowmelt into Lakes Łebsko and Gardno, and ultimately into the Baltic Sea. After World War II, the establishment of State Agricultural Farms and continued intensification of agriculture, especially livestock production, significantly increased demand for forage crops. As a result, many wetland areas were further transformed into moist and eventually dry meadows suitable for grazing. Peat extraction also expanded during the 20th century^21^. Over two centuries, intensive drainage development led to substantial wetland loss and degradation. Recognition of the ecological value of threatened peatland habitats, especially actively growing raised bogs, degraded bogs, and bog ecosystems with *Erica tetralix* L. (cross-leaved heath) and *Myrica gale* L. (bog myrtle), emerged in the second half of the 19th century. A major regional conservation milestone was the establishment of the Słowiński National Park in 1967, which encompassed extensive peatlands alongside coastal and dune habitats. In 1982, two peatland nature reserves were created: Torfowisko Pobłockie (121.31 ha) and Bagna Izbickie, initially covering 281.18 ha and expanded in 2009 to 847.51 ha. Active conservation measures in these reserves have focused on rewetting efforts, including the construction of sluices and small dams on drainage channels to retain precipitation within the peatland dome and raise water tables. Interventions have also involved the removal of encroaching woody vegetation^22^. Between 2016 and 2024, active management covered 62 ha of Bagna Izbickie. During this period, spontaneous tree regrowth was removed from non-forested peatland domes, and 106 water-retention baffles were constructed within the drainage channels (Fig. 1). According to habitat monitoring data, the conservation status of peatland habitat has not deteriorated in recent years. In fact, on three monitored plots, the structural and functional condition of the peatland has shown measurable improvement^23^.

## Data acquisition

**Fig. 2:**
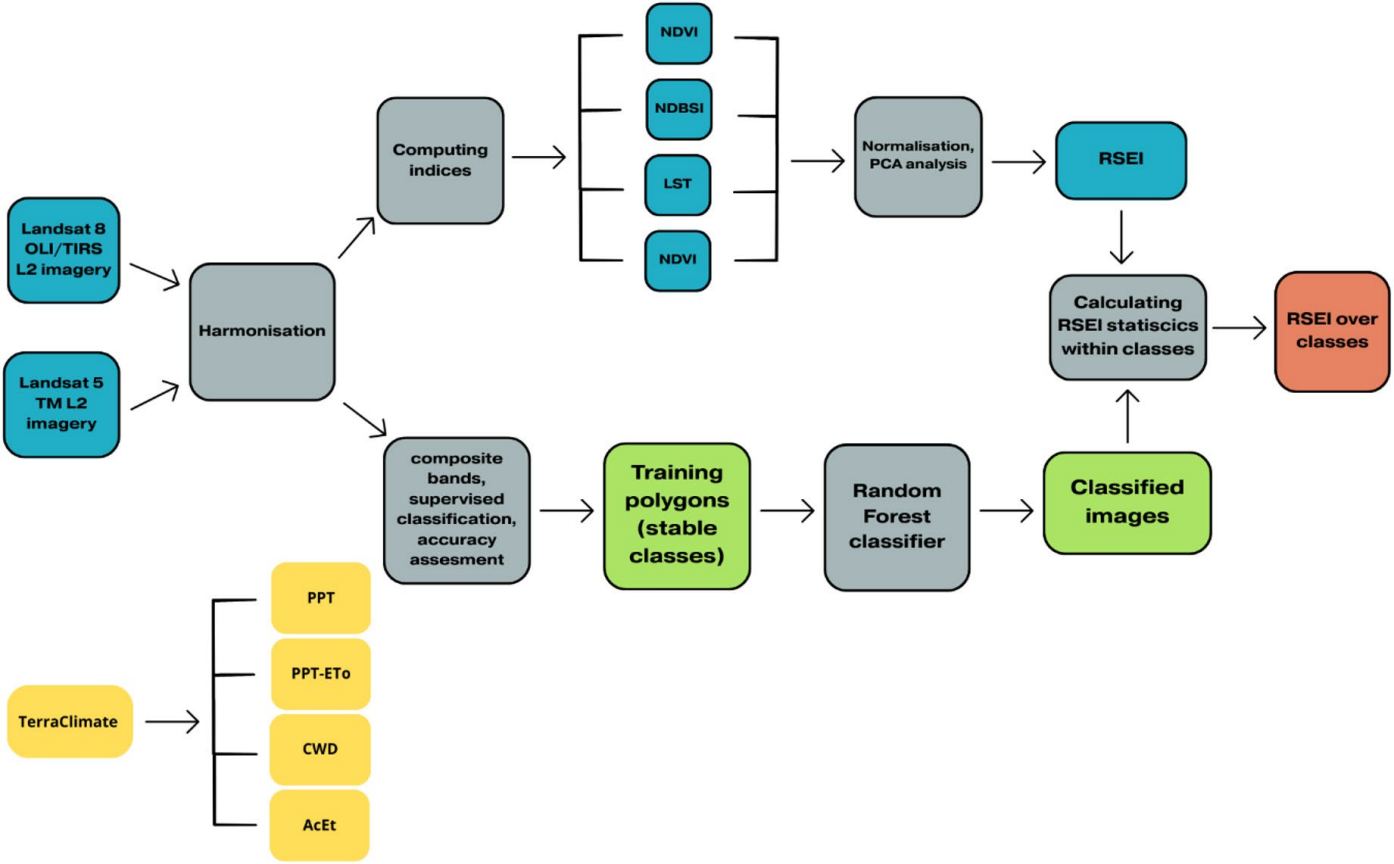
Study workflow.

The workflow applied in this study is presented in Figure. 2. Satellite and climatic datasets were acquired to characterise both land-cover dynamics and peatland ecological condition during 1984–2024 within the Bagna Izbickie Nature Reserve (ROI). Landsat imagery was sourced and processed using Landsat Collection 2 Level-2 (C2 L2) surface reflectance products, which provide atmospherically corrected reflectance suitable for multi-temporal analyses^24,25^. For the 1984–2010 period, Landsat 5 TM C2 L2 imagery was used. For 2011–2014 we additionally used Landsat 7 ETM+ Collection 2 Level-2 data; potential SLC-off gaps were mitigated through multi-scene compositing within the seasonal window, while for 2015–2024 Landsat 8 OLI C2 L2 imagery was used^24,26^. To maintain phenological consistency, imagery was restricted to the summer growing-season window, prioritising June acquisitions; where June-only selection yielded insufficient clear observations over the ROI, the window was extended to include July–August to ensure adequate sampling for annual composites, consistent with common practice in Landsat time-series studies^27,28^. The seasonal window was extended only when the June (or June–August) subset yielded insufficient valid observations after QA masking (i.e., too few cloud-free pixels to build a stable medoid composite).

Initial scene screening was performed using scene-level cloud cover metadata to exclude heavily contaminated acquisitions. Subsequently, pixel-level quality masking based on Landsat QA layers was applied to remove clouds, cirrus, cloud shadows, snow/ice, and fill pixels^25^. This ensured that only valid observations contributed to spectral indices, classification, and RSEI component derivation. The use of per-pixel cloud/shadow masking is a standard requirement for robust Landsat time series and change detection^29,30^. Pixel-level filtering used the QA_PIXEL and QA_RADSAT bands (cloud, shadow, snow/ice, fill, and radiometric saturation flags) included in Landsat C2 L2 products.

Hydroclimatic variables were obtained from the TerraClimate dataset via the ClimateEngine platform^31,32^. TerraClimate provides monthly data at a 1/24° (~ 4 km) spatial resolution. Variables included total precipitation (PPT), precipitation minus reference evapotranspiration (PPT–ETo), climatic water deficit (CWD), actual evapotranspiration (AcEt), and surface runoff, which together describe water supply and atmospheric demand relevant to peatland functioning^32^. Monthly values were extracted for the study period and aggregated to annual and June (and/or summer-season) summaries aligned with the satellite observation window. Descriptive statistics were calculated for all variables, and long-term tendencies were evaluated using linear regression as a transparent first-order assessment of directional change in hydroclimatic forcing^33^.

## Image classification

Land-cover mapping was conducted as a supervised, pixel-based classification workflow implemented in Google Earth Engine^34^. The landscape was classified into three categories: Forest, Bog and Grassland (encoded as integer classes to support temporal stacking and area calculations).

Landsat C2 L2 surface reflectance bands were harmonised to a common predictor set across sensors (Blue, Green, Red, NIR, SWIR1 and SWIR2), enabling consistent modelling across the Landsat archive^24,35^. In addition, indices capturing vegetation greenness and surface moisture were derived for each composite: NDVI^36,37^, NDMI^38^, NBR^39^ and MNDWI^40^. All predictors were calculated at 30 m resolution and clipped to the ROI. Cloud, shadow, and snow masking and saturation screening were applied using QA layers prior to compositing and index calculation^25,29,30^.

To reduce artefacts associated with scene-to-scene variability, annual composites were constructed for a consistent growing-season window (June–August, with conditional extension as described above). A medoid compositing approach was used, selecting the single observation most similar to the multiband median within the window, thereby avoiding synthetic spectra produced by per-band median mosaics. Time-series compositing is a recognised strategy for stabilising annual summaries in Landsat-based monitoring^24,41^. Medoid compositing helps minimize radiometric artefacts and avoids synthetic spectra, selecting an observed pixel that is most representative for the season.

Training data were generated in a two-stage procedure. First, eight reference-year composites at five-year intervals were classified using a maximum likelihood method to obtain an initial delineation of land-cover classes. Pixels whose class remained unchanged across the reference years were then identified as ‘stable’ and converted into class-specific training polygons. From these polygons, an equal number of random training points per class were generated to produce a balanced training dataset. Predictor values were sampled at training points for multiple reference years spanning the study period to improve robustness across time and sensor conditions, an approach commonly recommended for long-term land-cover mapping^35^.

A Random Forest classifier was then trained using the pooled, balanced samples. Random Forest is widely used in remote sensing classification due to its ability to model non-linear class boundaries and its strong performance in heterogeneous landscapes^42,43^. Model performance was evaluated using a hold-out split of the samples into training and validation subsets. Confusion matrices were used to derive overall accuracy and Cohen’s kappa, and class-specific User’s and Producer’s accuracies were reported following standard accuracy assessment guidance^44^. Training samples were randomly split into independent training and validation subsets using a uniform random column, assigning 75% of samples to model fitting and 25% to validation. To ensure balanced and memory-safe accuracy assessment, validation metrics were computed on a stratified subset of the validation data (1,500 samples per class; total *n* = 4,500) and summarized with a confusion matrix.

To suppress spurious year-to-year label fluctuations, a 5-year moving-window majority filter (pixel-wise mode in a multi-year neighborhood) was applied to the annual land-cover maps, a common post-processing strategy to reduce classification noise and temporal ‘flicker’ in categorical time series^35,45^. Class areas were estimated by summing pixel areas (ha) within the ROI for each class and year. To verify errors and omissions, definitions consistent with the accuracy assessment of land cover maps were adopted:

Type I (false positive / commission error) = 1 − UA (User’s Accuracy).

Type II (false negative / omission error) = 1 − PA (Producer’s Accuracy).

The F1-score was calculated as the harmonic mean of precision and recall, where precision = UA and recall = PA:

$$F1 = 2 \times \frac{{UA \times PA}}{{UA + PA}}$$

## Remote sensing ecological index

RSEI (Remote Sensing Ecological Index) is an integrated measure of environmental condition, constructed from remote sensing components describing key ecosystem characteristics; its strength lies in its simultaneous capture of multidimensional aspects of environmental condition and its ability to make comparisons across time and space^14^. In the original RSEI framework^14^,proposed an integrated remote-sensing ecological index that combines four indicators representing greenness (NDVI), wetness (WET from Tasseled Cap), heat (LST), and dryness/bare soil exposure (typically NDBSI), and then applies principal component analysis (PCA) to derive a synthetic ecological-quality gradient. In practice, the four indicators are first normalized, PCA is performed, and the first principal component (PC1) is used as the integrated index; depending on the sign convention, PC1 is inverted (e.g., 1 − PC1) so that higher values consistently indicate better ecological condition, and the result is finally rescaled to the [0, 1] range for intercomparison^14,47^. In this study, RSEI is defined according to the component approach and dimension reduction using the principal component method, in which the first principal component reflects the common variance of the four input indicators and is then normalized to the range [0,1]:

$${RSEI}_{0}=1-{PC}_{1}\left[f(NDVI,WET,NDBSI,LST)\right]$$

where f denotes the vector of standardized indicator values^14^.

The four components reflect the fundamental properties of land cover: greenery (NDVI), moisture (WET), heat (LST), and ground exposure/brightness (NDBSI/BSI). NDVI (Normalized Difference Vegetation Index) estimates the intensity and vitality of vegetation based on the contrast between red and near-infrared reflectance; high values are typical of healthy, dense vegetation, while low values are typical of degraded or vegetation-free land^36,37^. WET is a component of the Tasseled Cap transformation and is an indicator of cover moisture (soil and vegetation); its determination is based on a set of platform-specific coefficients (TM/OLI), which allows it to differentiate well between dry and wet areas^48,49^. LST (Land Surface Temperature), obtained from thermal channels, reflects the energy balance of the surface; elevated LST is often associated with exposed soil and reduced vegetation cover, while lower values are recorded over surfaces with high evapotranspiration or water^50,51^. NDBSI/BSI (Normalized Difference Bare Soil/Brightness Index) characterizes the proportion and ‘brightness’ of exposed ground through a combination of red, blue, NIR and SWIR bands; higher values indicate the dominance of soils and anthropogenic surfaces with high reflectance, typically after loss of vegetation cover^52^. Thanks to the integration of these components, RSEI is widely used in the assessment of degraded land regeneration and long-term monitoring of environmental changes, providing a comparable, scalable, and synthetic description of environmental quality^14,46^. Recent studies emphasize that this PCA-based construction can be sensitive to multi-temporal inconsistencies (sensor/atmospheric differences, phenology, and changing indicator loadings), which may introduce artificial fluctuations in long time series; accordingly, strengthened time-series implementations and bias-reduction strategies have been proposed^53^. Moreover, although RSEI has been widely adopted beyond urban contexts, its interpretation should remain process-aware and biome-specific; for example, in vegetation-transition systems, increasing greenness can elevate RSEI even when the underlying habitat integrity changes, underscoring the need for critical interpretation and, where possible, complementary diagnostics^54^.

Four input indicators were integrated using principal component analysis (PCA), and the first principal component (PC1) was used to construct the RSEI. PCA was performed independently for each year using the four normalized components. Due to different units and ranges of values, all indicators were normalized prior to analysis. PC1 synthesizes the common variability of NDVI, WET, NDBSI and LST, while the RSEI itself was calculated based on normalized values and scaled to the range [0, 1] (Table 1). LST was obtained from the Landsat Collection 2 Level-2 Surface Temperature product and converted to °C using the product scaling parameters. Higher values — closer to 1 — indicate better environmental quality. In practice, the RSEI was classified into five categories, every 0.2: level 1 (Poor): 0–0.2; level 2 (Satisfactory): 0.2–0.4; level 3 (Moderate): 0.4–0.6; level 4 (Good): 0.6–0.8; level 5 (Excellent): 0.8–1. The final RSEI value thus provides a synthetic description of the ecological status of the study area.

**Table 1:** Formulas for individual factors of the RSEI index.

Factor	Formula	Informations
WET	${\boldsymbol{\beta}}_{1}{\boldsymbol{B}}_{\boldsymbol{b}\boldsymbol{l}\boldsymbol{u}\boldsymbol{e}}+{\boldsymbol{\beta}}_{2}{\boldsymbol{B}}_{\boldsymbol{g}\boldsymbol{r}\boldsymbol{e}\boldsymbol{e}\boldsymbol{n}}+{\boldsymbol{\beta}}_{3}{\boldsymbol{B}}_{\boldsymbol{r}\boldsymbol{e}\boldsymbol{d}}+{\boldsymbol{\beta}}_{4}{\boldsymbol{B}}_{\boldsymbol{n}\boldsymbol{i}\boldsymbol{r}}+{\boldsymbol{\beta}}_{5}{\boldsymbol{B}}_{\boldsymbol{s}\boldsymbol{w}\boldsymbol{i}\boldsymbol{r}1}+{\boldsymbol{\beta}}_{6}{\boldsymbol{B}}_{\boldsymbol{s}\boldsymbol{w}\boldsymbol{i}\boldsymbol{r}2}$	${\boldsymbol{B}}_{\boldsymbol{b}\boldsymbol{l}\boldsymbol{u}\boldsymbol{e}}$, ${\boldsymbol{B}}_{\boldsymbol{g}\boldsymbol{r}\boldsymbol{e}\boldsymbol{e}\boldsymbol{n}}$, ${\boldsymbol{B}}_{\boldsymbol{r}\boldsymbol{e}\boldsymbol{d}}$, ${\boldsymbol{B}}_{\boldsymbol{n}\boldsymbol{i}\boldsymbol{r}}$, ${\boldsymbol{B}}_{\boldsymbol{s}\boldsymbol{w}\boldsymbol{i}\boldsymbol{r}1}$, ${\boldsymbol{B}}_{\boldsymbol{s}\boldsymbol{w}\boldsymbol{i}\boldsymbol{r}2}$ represent the reflectance coefficient in the Landsat 5/8 band, respectively; ${\boldsymbol{\beta}}_{\boldsymbol{i}}$ are the parameters of the Landsat 5/8 bands. SI and IBI denote the soil index and building index, respectively. T denotes the temperature of the bright surface. ${\boldsymbol{K}}_{1}$ i ${\boldsymbol{K}}_{2}$ are calibration parameters for surface temperature.
NDVI	${(\boldsymbol{B}}_{\boldsymbol{n}\boldsymbol{i}\boldsymbol{r}}-{\boldsymbol{B}}_{\boldsymbol{r}\boldsymbol{e}\boldsymbol{d}})/{(\boldsymbol{B}}_{\boldsymbol{n}\boldsymbol{i}\boldsymbol{r}}+{\boldsymbol{B}}_{\boldsymbol{r}\boldsymbol{e}\boldsymbol{d}})$	
NDBSI	$(\boldsymbol{S}\boldsymbol{I}+\boldsymbol{I}\boldsymbol{B}\boldsymbol{I})/2$	
SI	$[\left({\boldsymbol{B}}_{\boldsymbol{n}\boldsymbol{i}\boldsymbol{r}}+{\boldsymbol{B}}_{\boldsymbol{r}\boldsymbol{e}\boldsymbol{d}}\right)-\left({\boldsymbol{B}}_{\boldsymbol{n}\boldsymbol{i}\boldsymbol{r}}+{\boldsymbol{B}}_{\boldsymbol{b}\boldsymbol{l}\boldsymbol{u}\boldsymbol{e}}\right)]/[\left({\boldsymbol{B}}_{\boldsymbol{n}\boldsymbol{i}\boldsymbol{r}}+{\boldsymbol{B}}_{\boldsymbol{r}\boldsymbol{e}\boldsymbol{d}}\right)+\left({\boldsymbol{B}}_{\boldsymbol{n}\boldsymbol{i}\boldsymbol{r}}+{\boldsymbol{B}}_{\boldsymbol{b}\boldsymbol{l}\boldsymbol{u}\boldsymbol{e}}\right)]$	
IBI	$\{2{\boldsymbol{B}}_{\boldsymbol{s}\boldsymbol{w}\boldsymbol{i}\boldsymbol{r}2}/({\boldsymbol{B}}_{\boldsymbol{s}\boldsymbol{w}\boldsymbol{i}\boldsymbol{r}1}+{\boldsymbol{B}}_{\boldsymbol{n}\boldsymbol{i}\boldsymbol{r}})-[{\boldsymbol{B}}_{\boldsymbol{n}\boldsymbol{i}\boldsymbol{r}}/({\boldsymbol{B}}_{\boldsymbol{r}\boldsymbol{e}\boldsymbol{d}}+{\boldsymbol{B}}_{\boldsymbol{n}\boldsymbol{i}\boldsymbol{r}})+{\boldsymbol{B}}_{\boldsymbol{g}\boldsymbol{r}\boldsymbol{e}\boldsymbol{e}\boldsymbol{n}}/({\boldsymbol{B}}_{\boldsymbol{s}\boldsymbol{w}\boldsymbol{i}\boldsymbol{r}1}+{\boldsymbol{B}}_{\boldsymbol{g}\boldsymbol{r}\boldsymbol{e}\boldsymbol{e}\boldsymbol{n}})]/\{{2\boldsymbol{B}}_{\boldsymbol{s}\boldsymbol{w}\boldsymbol{i}\boldsymbol{r}2}/({\boldsymbol{B}}_{\boldsymbol{s}\boldsymbol{w}\boldsymbol{i}\boldsymbol{r}1}+{\boldsymbol{B}}_{\boldsymbol{n}\boldsymbol{i}\boldsymbol{r}})+[{\boldsymbol{B}}_{\boldsymbol{n}\boldsymbol{i}\boldsymbol{r}}/({\boldsymbol{B}}_{\boldsymbol{r}\boldsymbol{e}\boldsymbol{d}}+{\boldsymbol{B}}_{\boldsymbol{n}\boldsymbol{i}\boldsymbol{r}})+{\boldsymbol{B}}_{\boldsymbol{g}\boldsymbol{r}\boldsymbol{e}\boldsymbol{e}\boldsymbol{n}}/({\boldsymbol{B}}_{\boldsymbol{s}\boldsymbol{w}\boldsymbol{i}\boldsymbol{r}1}+{\boldsymbol{B}}_{\boldsymbol{g}\boldsymbol{r}\boldsymbol{e}\boldsymbol{e}\boldsymbol{n}}\left)\right]$	
LST	$\boldsymbol{T}/\left[1+\left(\boldsymbol{\lambda}\boldsymbol{T}/\boldsymbol{\rho}\right)\boldsymbol{l}\boldsymbol{n}\boldsymbol{\epsilon}\right]-273.15$	
T	${\boldsymbol{K}}_{2}/\boldsymbol{l}\boldsymbol{n}({\boldsymbol{K}}_{1}/{\boldsymbol{B}}_{\boldsymbol{s}\boldsymbol{w}\boldsymbol{i}\boldsymbol{r}1}+1)$	

Long-term trends in RSEI and its components (ROI-level and class-stratified summaries) were quantified using the Theil-Sen slope estimator, which is robust to outliers and non-normal data^55,56^. Trend significance was assessed using the non-parametric Mann–Kendall test and reported as Kendall’s τ with two-sided p-values^57^. Theil–Sen 95% confidence intervals were derived using the rank-based approach associated with Sen’s slope estimator^56^. Because serial correlation can inflate the Type I error rate of Mann-Kendall trend tests, we evaluated lag-1 autocorrelation in the annual time series; if significant autocorrelation was detected, we would apply an autocorrelation-robust variant (e.g., variance correction or trend-free prewhitening)^58,59^. Statistical significance was evaluated at α = 0.05.

## Results

### Hydroclimate background

**Fig. 3:**
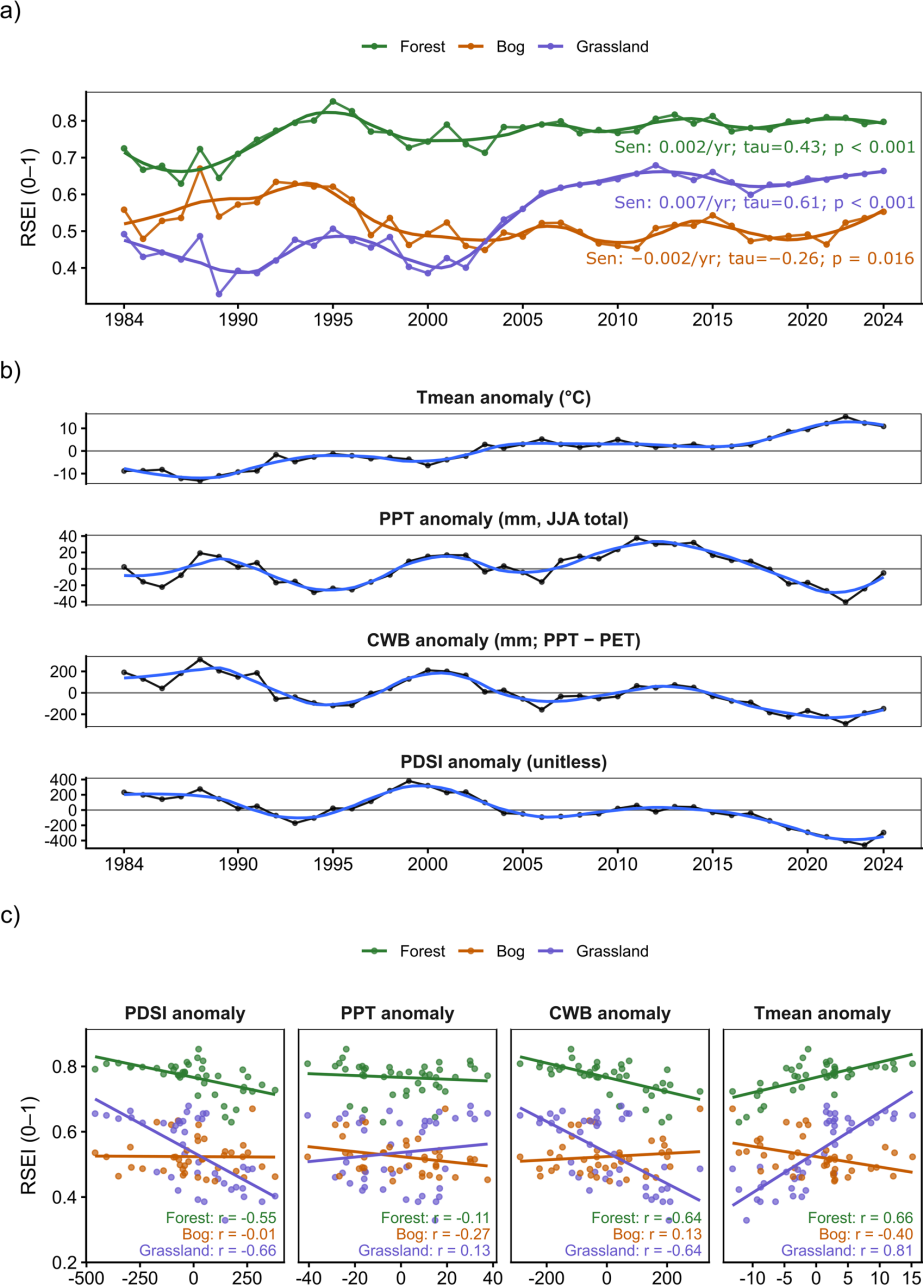
Hydroclimate variability and class-specific RSEI responses to summer climate anomalies (1984–2024).

Across the study period (1984–2024), the hydroclimatic background shows a clear warming signal accompanied by a progressive drying tendency in recent decades (Fig. 3b). The summer (JJA) mean temperature anomaly increased steadily from predominantly negative values in the mid-1980s to persistently positive anomalies after the early 2000s, with the highest values occurring in the late 2010s-early 2020s. In contrast, precipitation anomalies displayed pronounced interannual to decadal variability, with wetter phases around 1998–2002 and 2010–2013 and drier conditions dominating after 2015, culminating in strongly negative precipitation anomalies around 2021–2023. The climatic water balance (CWB = PPT - PET) closely tracked these shifts and indicates a sustained decline in summer water availability after 2014, reaching markedly negative values in the early 2020s. Consistent with this pattern, PDSI anomalies transitioned from comparatively favourable conditions in the late 1990s and early 2000s toward increasingly negative values during the last decade of the record, highlighting an intensification of summer moisture deficits.

These hydroclimatic dynamics provide an essential context for interpreting class-specific trajectories in the remote-sensing ecological index (Fig. 3a). Forest exhibited consistently high RSEI values throughout the record and a modest but significant positive long-term trend (Theil-Sen slope 0.002 yr⁻¹; Kendall’s τ = 0.43; *p* < 0.001), indicating a gradual improvement in the integrated greenness-moisture-heat-brightness signal. Grassland showed the strongest positive RSEI trajectory, particularly after the early 2000s, resulting in a significant upward trend (0.007 yr⁻¹; τ = 0.61; *p* < 0.001). In contrast, bog RSEI declined slightly over time (-0.002 yr⁻¹; τ = -0.26; *p* = 0.016), with lower values during the warm and hydroclimatically drier late-2010s and early-2020s period.

The relationships between RSEI and hydroclimate further underscore the importance of temperature-driven constraints on habitat condition during summer (Fig. 3c). In forest and grassland, RSEI was positively associated with summer temperature anomalies (*r* = 0.66 and *r* = 0.81, respectively), whereas bog showed an inverse relationship (*r* = -0.40), indicating contrasting thermal sensitivities among land-cover classes. Moisture-related metrics exhibited similarly divergent patterns: RSEI was negatively correlated with PDSI and CWB anomalies in forest and grassland (PDSI: *r* = -0.55 and − 0.66; CWB: *r* = -0.64 and − 0.64), while bog responses were weak to modestly positive (PDSI: *r* = -0.01; CWB: *r* = 0.13). Precipitation anomalies showed comparatively weaker and more variable associations overall (forest *r* = -0.11; bog *r* = -0.27; grassland *r* = 0.13). The hydroclimatic record indicates that the recent warming and declining summer water balance coincided with a divergence in RSEI responses, with grassland and forest showing net improvements under warmer summers, while the bog class exhibited an opposing temperature response and a slight long-term decline.

## Land-cover change

**Fig. 4:**
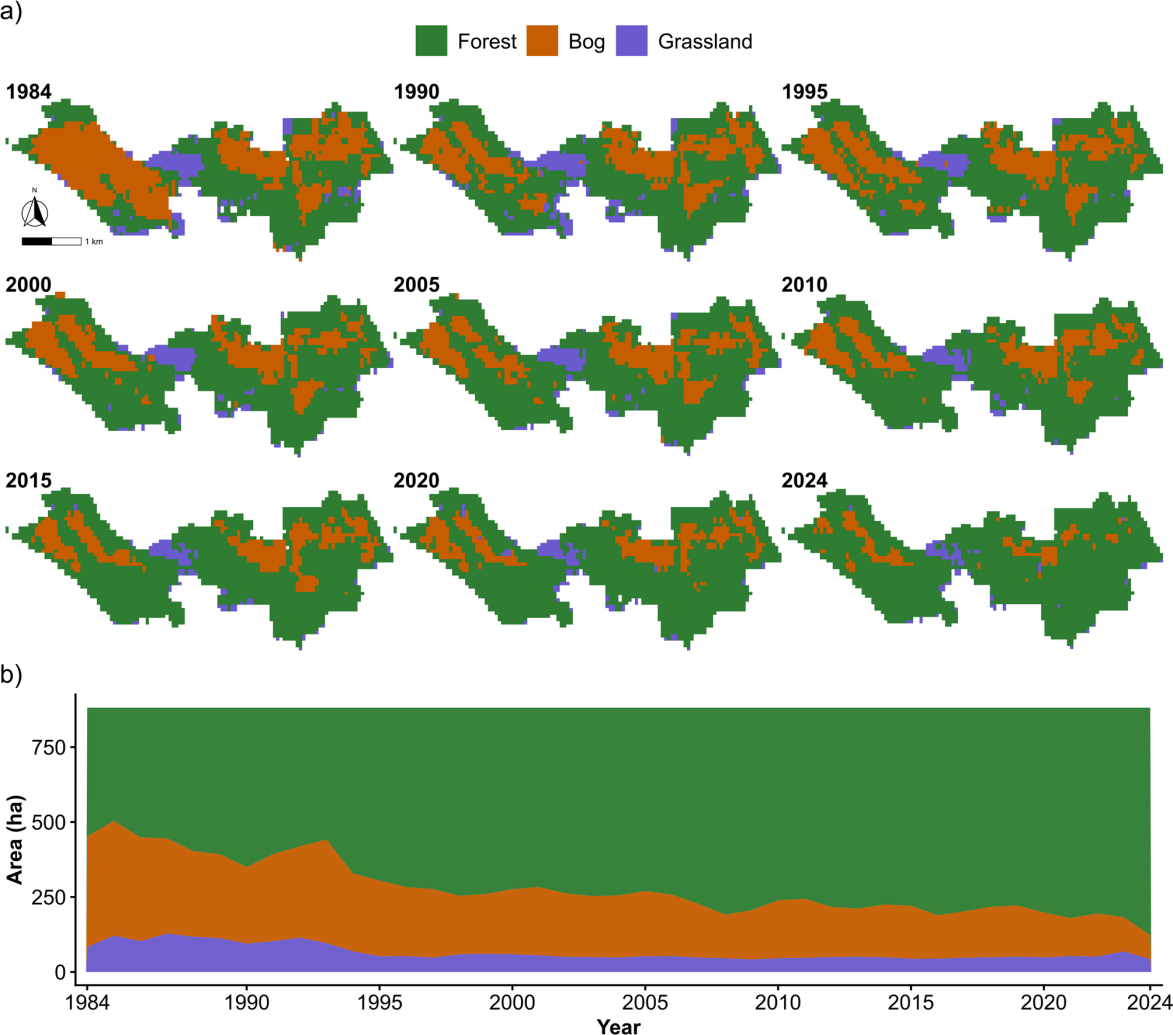
Spatiotemporal changes in land-cover classes and their areal trajectories.

The land-cover maps reveal a pronounced reconfiguration of the Bagna Izbickie landscape over the last four decades, dominated by a net expansion of forest at the expense of bog and, to a lesser extent, grassland (Fig. 4a). In 1984, forest already represented the largest class (461.8 ha; 52.39%), followed by bog (327.9 ha; 37.19%) and grassland (91.8 ha; 10.41%) (Table 2). Over time, forest progressively increased and became more spatially continuous, particularly along margins and within sectors that were previously bog-dominated, indicating sustained woody encroachment or successional change in formerly open wetland areas. This transition is visible in the mapped snapshots, where bog becomes increasingly fragmented and restricted to smaller patches, while grassland remains confined to localized areas with comparatively limited areal extent (Fig. 4a).

**Table 2:** Areal extent of land-cover classes in the study area (ha and %), selected years (1984–2024).

Year	Forest	Bog	Grassland		Forest	Bog	Grassland
	(ha)				(%)		
1984	461.8	327.9	91.8		52.39	37.19	10.41
1990	519.0	254.1	108.3		58.88	28.83	12.29
1995	565.8	259.0	56.7		64.18	29.38	6.43
2000	591.9	220.7	68.9		67.14	25.04	7.82
2005	592.3	224.7	64.5		67.19	25.49	7.32
2010	626.3	199.9	55.3		71.05	22.68	6.27
2015	641.6	187.8	52.1		72.78	21.31	5.91
2020	663.0	162.8	55.7		75.21	18.47	6.32
2024	738.4	89.8	53.3		83.77	10.19	6.04

The area time series corroborates these spatial patterns and quantifies their magnitude (Fig. 4b). Forest area increased steadily from 461.8 ha (52.39%) in 1984 to 663.0 ha (75.21%) in 2020, and reached 738.4 ha (83.77%) by 2024. In parallel, bog area declined sharply from 327.9 ha (37.19%) in 1984 to 162.8 ha (18.47%) in 2020 and further to 89.8 ha (10.19%) in 2024, representing a net loss of 238.1 ha (-27.0%). Grassland occupied a minor fraction of the landscape throughout the record and showed comparatively modest variability: it peaked at 108.3 ha (12.29%) in 1990, then decreased and stabilized around 52–56 ha (5.9–6.3%) in 2010–2024 (e.g., 55.7 ha; 6.32% in 2020 and 53.3 ha; 6.04% in 2024). Overall, these trajectories indicate a long-term shift from a more open wetland complex toward a forest-dominated land cover, with substantial contraction and fragmentation of bog habitats at the landscape scale (Fig. 4).

**Table 3:** Class-specific commission (Type I) and omission (Type II) errors, F1-score (mean ± SD) and Markov pixel transition probability across the study period.

Class	Type I (commission)	Type II (omission)	F1-score		Class transition probability to:			
					Bog		Forest	Grassland
Bog	0.248 ± 0.076	0.139 ± 0.097	0.801 ± 0.073			0.1561	0.7942	0.0497
Forest	0.029 ± 0.029	0.153 ± 0.053	0.904 ± 0.031			0.0019	0.9222	0.0760
Grassland	0.386 ± 0.195	0.000 ± 0.000	0.747 ± 0.138			0.0005	0.5478	0.4518

The accuracy analysis (Table 3) showed that for the forest class, Type I error was practically absent, indicating that the probability of the algorithm assigning another class when forest actually occurs in a given area is very low, with a mean value of 0.029. For bog and grassland, Type I error occurred more frequently, with mean shares of 0.248 and 0.386, respectively. Type II error occurred only for bog and forest, with similar frequencies (mean values of 0.139 and 0.153, respectively), while no Type II error was observed for grassland. The overall F1 score indicates high classification accuracy for forest, with a mean value of 0.904, and the lowest classification accuracy for grassland, with a mean value of 0.747. The analysis of class transition probabilities (Table 3) revealed an almost zero probability of forest or grassland transitioning into bog, while the probability of bog persisting throughout the entire period was 0.1561. The forest class showed high stability, with a persistence probability of 0.9222, and a relatively high probability of grassland (0.5478) and bog (0.7942) transitioning into forest. Cohen’s Kappa for supervised classification ranged from 0.610 to 0.875 with a mean of 0.723.

## RSEI trajectories and class differences

**Fig. 5:**
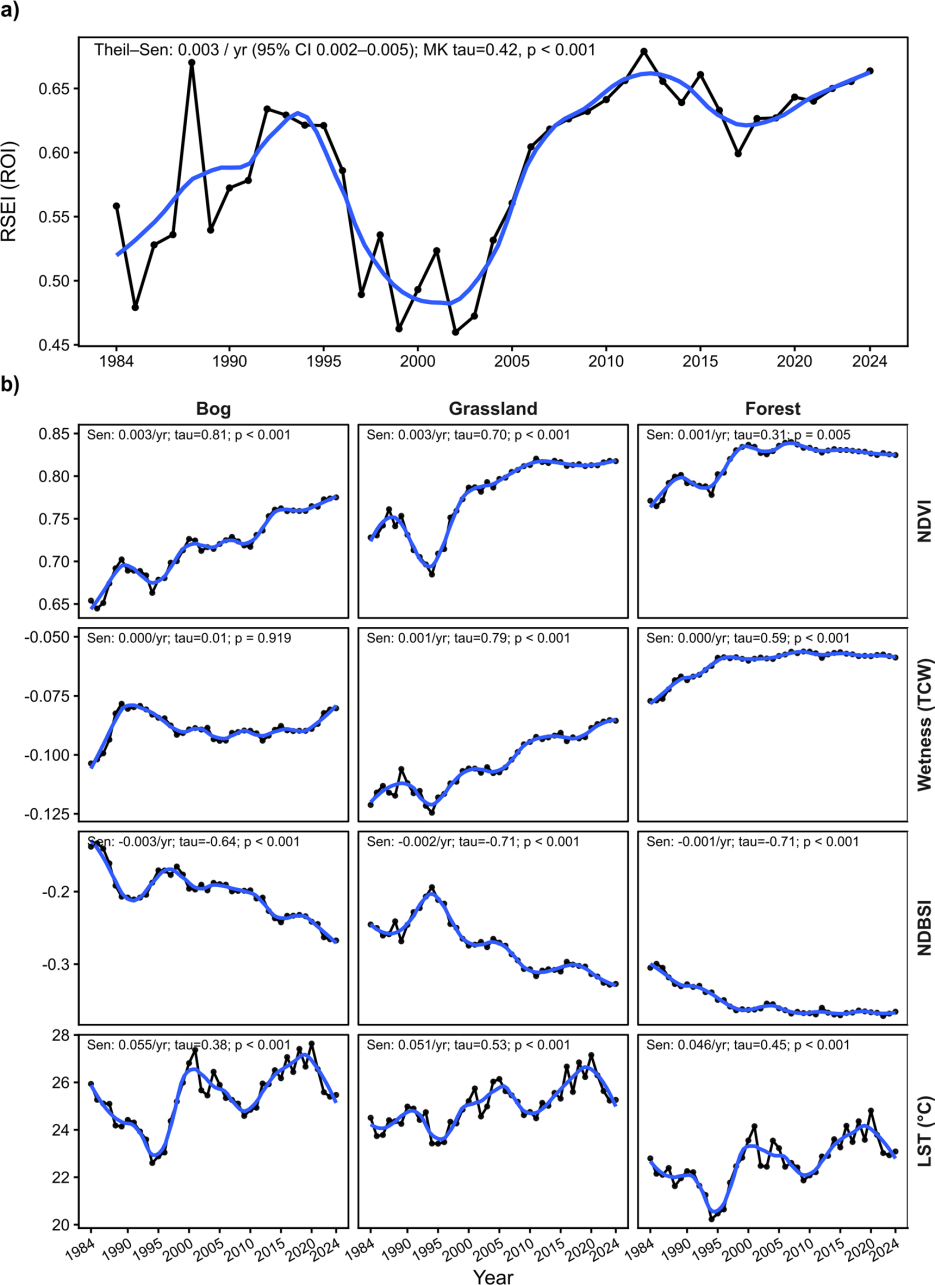
Long-term RSEI trajectories and component trends across land-cover classes (1984–2024).

The ecological condition of the study area, expressed as the ROI-level RSEI, exhibited a significant long-term increase over the full observation period (1984–2024; Fig. 5a). The Theil-Sen estimate indicated a positive monotonic trend of 0.003 yr^− 1^ (95% CI 0.002–0.005), supported by a significant Mann-Kendall statistic (τ = 0.42, *p* < 0.001). Superimposed on this long-term trajectory, the time series showed pronounced multi-year variability: RSEI rose through the late 1980s and early 1990s, declined to a minimum around the late 1990s and early 2000s, and then increased sharply after 2004–2006, reaching sustained higher values during the 2010s and early 2020s.

Component-level trends revealed consistent yet class-dependent pathways underlying these trajectories (Fig. 5b). Greenness (NDVI) increased across all land-cover classes, with the strongest monotonic signal in bog and grassland (both Sen = 0.003 yr^− 1^, τ = 0.81 and 0.70, respectively; *p* < 0.001), while forest showed a smaller but still significant increase (Sen = 0.001 yr^− 1^, τ = 0.31, *p* = 0.005). In contrast, the moisture-related component (Wetness, TCW) differentiated the classes: grassland displayed a clear increase (Sen = 0.001 yr^− 1^, τ = 0.79, *p* < 0.001) and forest also increased significantly despite a near-zero slope (τ = 0.59, *p* < 0.001), whereas bog wetness showed no monotonic trend (Sen = 0.000 yr^− 1^, τ = 0.01, *p* = 0.919). The soil exposure proxy (NDBSI) decreased consistently in all classes (bog − 0.003 yr⁻¹, grassland − 0.002 yr^− 1^, forest − 0.001 yr^− 1^; τ from − 0.64 to -0.71, all *p* < 0.001), indicating a long-term reduction in exposed surfaces and increasing canopy and vegetation cover. At the same time, LST increased in every class with comparable magnitudes (0.046–0.055 °C yr^− 1^, τ = 0.38–0.53, all *p* < 0.001), consistent with the broader warming context observed in the hydroclimatic record.

These patterns indicate that the overall improvement in ROI-level RSEI coincided with pervasive greening (increase of NDVI) and declining surface exposure (decrease of NDBSI) across all classes, while the wetness signal diverged among habitats strengthening in grassland and forest but remaining statistically unchanged in bog. The bog class experienced strong greening without a corresponding increase in wetness, whereas grassland and forest combined greening with increasing wetness, suggesting different ecological trajectories despite a shared background of rising summer surface temperature.

### Spatial pattern

**Fig. 6:**
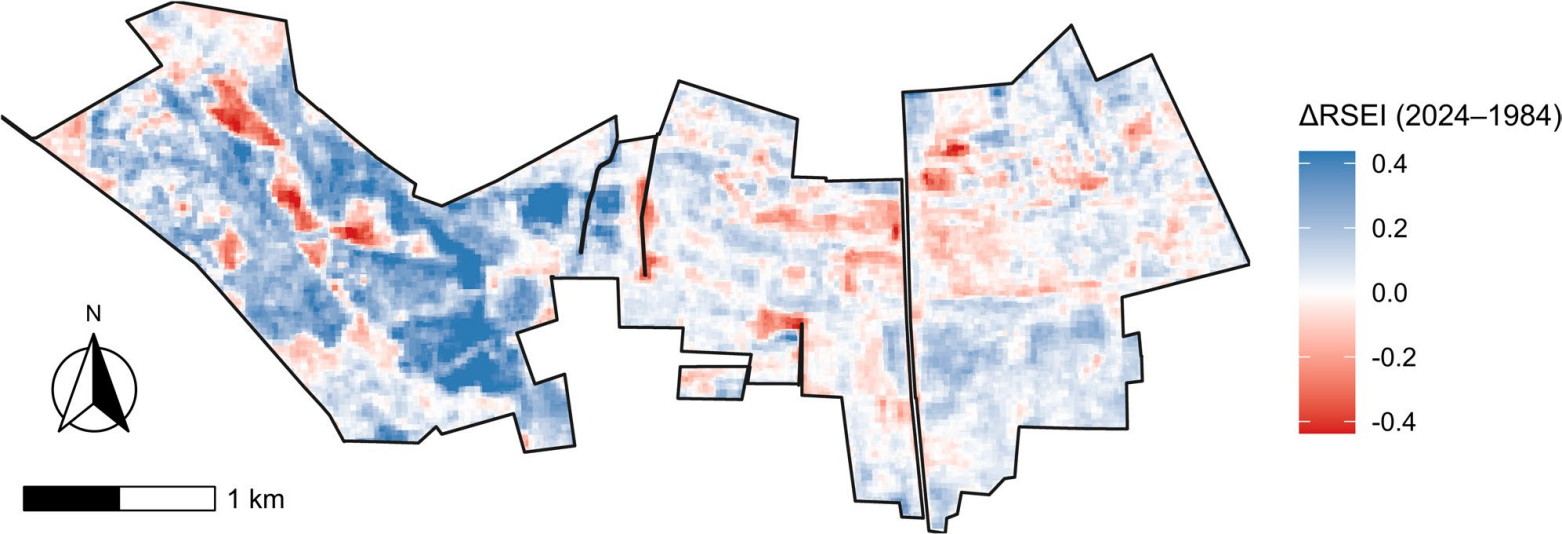
Spatial heterogeneity of ecological-condition change across the study area inferred from ΔRSEI.

The spatial distribution of long-term change in ecological condition (ΔRSEI = RSEI_2024_ - RSEI_1984_) was highly heterogeneous but clearly structured, indicating that improvement and degradation were not randomly dispersed across the landscape (Fig. 6). Overall, ΔRSEI was skewed towards positive values, with a mean of 0.073 and a median of 0.053, while the interquartile range (IQR = 0.176) demonstrates substantial within-site variability. Most pixels experienced modest changes (10th-90th percentiles: -0.090 to 0.282), whereas the tails contained localized extremes (2nd-98th percentiles: -0.230 to 0.437; absolute range: -0.536 to 0.911), consistent with small areas of strong deterioration or strong improvement embedded within broader regions of moderate change.

The map highlights coherent zones of improvement (blue) interspersed with discrete clusters of degradation (red), with the strongest positive changes forming broad, spatially continuous patches, particularly across large parts of the western sector of the site, whereas negative changes were more localized and patchy (Fig. 6). Using a data-driven threshold for change, the area exhibiting detectable long-term change comprised 847.7 ha. Within this subset, improvements predominated: 584.0 ha (68.9%) showed positive ΔRSEI, while 263.7 ha (31.1%) showed negative ΔRSEI. Patch-based summaries further indicate that meaningful changes tend to aggregate into a small number of large contiguous patches, with the largest patch reaching 560 ha, underscoring the landscape-scale character of the dominant change processes.

The long-term shift in RSEI was not uniform across Bagna Izbickie, but instead occurred as large, spatially coherent improvement zones coupled with localized degradation hotspots, a pattern consistent with spatially structured drivers (e.g., land-cover transitions, hydrological gradients, and management legacies) acting at scales larger than individual pixels (Fig. 6).

## Discussion

Our Landsat-based reconstruction reveals a clear, directional transformation of the Bagna Izbickie landscape over 1984–2024, characterized by a strong expansion of forest cover and a marked contraction of open bog. Forest increased from 461.8 ha (52.39%) in 1984 to 738.4 ha (83.77%) in 2024, whereas bog decreased from 327.9 ha (37.19%) to 89.8 ha (10.19%), representing a net loss of 238.1 ha of bog-class area. Such a shift from an open wetland mosaic toward a forest-dominated matrix is consistent with well-documented degradation pathways in raised bogs, where historical drainage and subsequent lowering of the water table remove the primary constraint on tree recruitment and survival. In a Baltic raised bog in north-western Poland, drainage ditches were shown to enhance forest succession and accelerate woody expansion into mire surfaces^60^. Comparable processes have also been reported for drained peat bog systems in western Poland, where woody encroachment transforms habitat conditions and drives the retreat of specialist bog species^61^. In our case, the progressive spatial continuity of the forest and the shrinking extent of the bog class imply not only habitat loss but also increasing fragmentation of remaining open patches, a configuration that can further amplify edge-driven successional feedbacks and reduce the resilience of open bog vegetation.

The complex of peat bogs analysed in the study forms a fine-grained mosaic of wetlands, grasslands, and forests, where ecotones are common and class boundaries can be inherently diffuse. At Landsat spatial resolution (30 m), mixed pixels at habitat edges are therefore unavoidable, which is a well-recognized limitation in long-term wetland monitoring based on the Landsat archive and pixel-based classifications^62^. In such mosaics, apparent transitions between “bog-grassland-forest” may partly reflect sub-pixel mixing and seasonal variability in moisture and canopy cover rather than fully discrete habitat shifts. This issue is particularly relevant for drained or drying raised bogs, where woody encroachment can accelerate and progressively alter spectral responses. In Polish raised bogs, drainage infrastructure has been shown to facilitate forest succession and tree expansion into mire surfaces^60^. Comparable evidence from Lithuania demonstrates that tracking peatland degradation and tree encroachment requires clear class separation in long-term monitoring^63^. Otherwise, gradual transitions may be obscured and vegetation-condition trends can be misinterpreted. Consequently, robust post-classification accuracy assessment is essential to quantify uncertainty, especially along ecotones where the probability of confusion is highest, and to ensure that long-term trends are interpreted as ecological change rather than classification noise^62^. Where feasible, integrating complementary sensors (e.g., optical + SAR) can further reduce ambiguity in peatland wetness and boundary dynamics and improve long-term monitoring reliability^64^. Classification errors (Type I) and omissions (Type II) indicate that areas classified on the map, as well as any summaries stratified by classes, may be subject to bias, since misclassification directly affects both area estimates and statistics calculated within classes. In particular, confusion between bogs and grasslands may lead to under- or overestimation of bog area and may systematically alter class-based RSEI trajectories, even when overall accuracy remains high^65^. Therefore, changes and trends were interpreted with caution and emphasized only where they appeared robust to the observed class-specific error structure, while the remaining uncertainty reflects the expected sensitivity of land-use change inference to classification errors and omissions in thematic maps^66^.

The hydroclimatic context provides a mechanistic backdrop to these land-cover trajectories. Our TerraClimate-based analyses indicate a warming signal and an intensifying tendency toward summer moisture limitation, expressed through changes in climatic water balance and drought-relevant metrics. This pattern is aligned with broader assessments that emphasize the role of increasing atmospheric evaporative demand under warming, which can exacerbate agricultural and ecological drought even where precipitation trends are ambiguous^67^. For ombrotrophic raised bogs, which rely primarily on precipitation inputs and are tightly governed by near-surface water-table dynamics, such changes in summer water deficit are particularly consequential. TerraClimate is well suited to quantify this kind of regional hydroclimatic pressure because it provides internally consistent monthly climate and climatic water-balance variables at relatively fine spatial resolution and has become a widely used forcing dataset for ecohydrological studies^32^. This hydroclimatic pressure is also reflected in the satellite-derived surface thermal signal: LST increased significantly across all land-cover classes (Sen = + 0.055 °C yr^− 1^ in bog; +0.051 °C yr^− 1^ in grassland; +0.046 °C yr^− 1^ in forest; all *p* < 0.001), consistent with progressive surface warming. At the same time, the wetness-related component shows no monotonic trend in bog pixels (TCW Sen = 0; τ = 0; *p* = 0.919), indicating that greening did not translate into a sustained increase in surface wetness in the remaining bog patches. TerraClimate should be interpreted as a regional constraint rather than a direct surrogate for peatland water-table depth, which is additionally shaped by ditch networks, peat hydraulic properties, and microtopography.

A central result of this study is that remote sensed ecological condition trajectories are strongly class-dependent and can diverge from the direction of habitat change when summarized at the landscape scale. At the ROI level, RSEI shows a significant upward trend (Theil-Sen + 0.003 yr^− 1^), yet this aggregated signal masks contrasting behavior among land-cover classes. Forest exhibits consistently high RSEI values and a modest but significant increase (+ 0.002 yr^− 1^), grassland shows the strongest increase (+ 0.007 yr^− 1^), while bog RSEI declines slightly but significantly (-0.002 yr^− 1^) and reaches lower values during the warm and hydroclimatically drier late-2010s to early-2020s period. This split matters because RSEI-type indices are designed to synthesize multiple surface properties (greenness, wetness, dryness, and heat) into a composite proxy of surface eco-environmental quality^46,68^. RSEI also inherits limitations from its PCA-based construction^54,69^. In landscapes undergoing peatland-to-forest transition, the same processes that represent peatland degradation (woody encroachment and canopy densification) can increase greenness and reduce exposed-soil signals, thereby raising the composite index even while the integrity of open bog habitat declines. In other words, an improving ROI-level RSEI is not evidence of peatland recovery; it can be an expected by-product of forest expansion in a composite metric that rewards canopy cover.

Peatlands are highly dynamic hydro-ecological systems, and this has implications for the interpretation and scientific soundness of generic composite indices such as RSEI. Conceptually, RSEI was designed as a multivariate surface “eco-environment” proxy integrating greenness, wetness, heat, and dryness, usually via a PCA-based synthesis. While this is useful for broad-scale screening, recent methodological work shows that conventional RSEI can suffer from multi-temporal instability (e.g., time-varying component loadings, phenology or sensor effects) that may introduce bias or artificial fluctuations in long time series; time-series explicit variants (e.g., ts-RSEI) and continuity-oriented frameworks (e.g., CRSEI) have therefore been proposed to improve comparability through time^53,70^. Moreover, in peatlands undergoing woody encroachment, RSEI may increase due to rising greenness and reduced bare-soil signal even when the hydrological integrity of open bog habitat declines, which calls for process-aware interpretation and, ideally, peatland-specific augmentation^71^.

To improve the sensitivity of monitoring in hydrologically controlled systems, the RSEI-based framework should be supplemented with additional biophysical parameters that are more dependent on the water regime of peatlands and the structural characteristics of vegetation. A key example is the integration of modeled LAI, surface or soil moisture, and surface roughness, which were shown to provide higher sensitivity and precision when jointly derived from microwave (SAR) and optical observations and supported by field measurements^72^. This direction is strongly supported by recent peatland remote-sensing studies demonstrating that SAR-optical fusion can improve inference on water-table depth and wetness dynamics^64,73^. Practically, such augmentation could be implemented by coupling Landsat-based RSEI with Sentinel-1 SAR backscatter or metrics as moisture and roughness proxies and with optical vegetation-structure descriptors (e.g., LAI-related products or proxies), followed by either a modified multivariate index (e.g., adding a hydrology-focused component) or a multi-indicator dashboard where RSEI is interpreted alongside explicit wetness/structure variables^68,71^.

Component-level trends support this interpretation. NDVI increased in all classes, with the strongest trends in bog and grassland (both Sen = + 0.003 yr^− 1^; τ = 0.81 and 0.70; *p* < 0.001), while the forest NDVI trend was smaller (Sen = + 0.001 yr^− 1^; τ = 0.31; *p* = 0.005). Both changes mechanically push RSEI upward. Within peatland pixels, spatial differences in NDVI among patches may reflect not only vegetation densification, but also hydrological variation, including groundwater table depth, as well as the local expansion of bog heather (Erica tetralix L.) observed in the reserve. Because NDVI typically exerts a strong influence on the overall RSEI value^54^, increases in vascular cover can raise the composite index without implying hydrological recovery of the bog surface; peatland water stress and moisture conditions are often better captured by metrics exploiting NIR and SWIR information^74,75^, represented in the RSEI framework primarily through the Wetness-related component. In contrast, the moisture-oriented component (Tasseled Cap Wetness; TCW) differentiates habitats: grassland and forest show increasing wetness signals, whereas bog wetness shows no monotonic trend (Sen = 0; τ = 0; *p* = 0.919) and exhibits pronounced interannual variability. This result is consistent with a trajectory in which vegetation densifies (including woody encroachment) without hydrological recovery of the bog surface. Moisture-sensitive transformations such as Tasseled Cap Wetness were developed explicitly to yield physically interpretable axes related to brightness, greenness, and wetness in Landsat data^48^, and are therefore often more directly interpretable for wetland moisture conditions than composite indices optimized for general eco-environment assessment. More broadly, peatland remote sensing syntheses emphasize that optical moisture proxies can be informative but are sensitive to vegetation structure and pixel mixing in heterogeneous bog mosaics, reinforcing the need to interpret moisture signals in a land-cover-stratified way rather than relying on a single composite indicator^11^.

The contrasting thermal sensitivity across classes further supports the interpretation that warming exerts qualitatively different constraints on bog surfaces than on forest or grassland. In our results, RSEI is positively associated with summer temperature anomalies in forest and grassland (*r* = 0.66 and 0.81) but negatively associated with summer temperature anomalies in bog (*r* = -0.40). This sign reversal is ecologically plausible: forests and grasslands may experience productivity gains during warmer summers up to the point where water limitation dominates, whereas ombrotrophic bog surfaces are acutely constrained by near-surface moisture and can experience rapid deterioration during warm periods as evaporative demand rises. The IPCC assessment explicitly notes that increased atmospheric evaporative demand increases plant water stress and contributes to ecological drought^67^, which is consistent with the negative temperature-condition relationship we observe in bog pixels and with the role of summer deficits as a key stressor for peatland surface functioning.

From a conservation and management perspective, the combination of strong bog-area contraction and class-specific RSEI decline in bog pixels suggests that local improvements achieved through active management may not yet be sufficient to counteract broader landscape-scale drivers. Actions such as ditch blocking and removal of woody regrowth are widely used in peatland restoration, but their effects can be spatially uneven and potentially outweighed by legacy drainage networks and increasing summer deficits if interventions are limited in extent or not hydrologically comprehensive. Our findings therefore point to a practical implication: monitoring and evaluation should separate (i) land-cover transitions that directly reflect peatland integrity (extent and fragmentation of open bog) from (ii) composite surface condition metrics that can be dominated by forest expansion. In this context, the most defensible role for RSEI in raised bog studies is as a complementary indicator of integrated surface condition, explicitly stratified by land-cover class, rather than as a stand-alone proxy for peatland status^46,68^.

To increase applied relevance, our Landsat-based products (annual land-cover maps, change trajectories, and class-stratified condition metrics) can be aligned with national wetland or peatland monitoring initiatives. POLWET, designed as a remote-sensing information system for monitoring and managing Ramsar wetlands in Poland through standardized satellite products and repeatable workflows, provides a natural framework for uptake^76^. Our approach adds multi-decadal, spatially explicit trajectories of habitat transitions (e.g., bog to grassland or forest) and uncertainty-aware, class-specific trends that support prioritizing intervention zones (e.g., accelerated woody encroachment or persistent wetness decline) beyond site-averaged indicators. These outputs are interoperable with the Geoserwis GDOŚ platform (WMS/WFS), enabling overlay and hotspot querying within legally protected boundaries^77^. They are also relevant to ongoing LIFE project implementation in Poland, by providing an objective baseline to evaluate whether interventions reduce drying-driven habitat conversion and improve peatland integrity. More broadly, syntheses stress that operational peatland monitoring benefits from scalable remote-sensing time series integrated into national decision-support systems, provided interpretation remains process-aware and habitat-stratified^11^.

Several limitations frame the interpretation of these results. First, the 30 m Landsat resolution necessarily mixes microforms typical of raised bogs (hummocks, hollows, small pools, shrub patches), which can blur boundaries between open bog, shrub/bog woodland, and forest, especially along edges and drainage features. This is a common constraint in long-term wetland monitoring, where the Landsat archive is uniquely valuable for multi-decadal trajectories but limited in resolving fine-grained wetland structure^62^. Second, while TerraClimate provides robust regional hydroclimatic context, it cannot capture site-specific hydrological behavior controlled by ditch networks, peat properties, and microtopography^32^. Third, composite indices like RSEI are sensitive to the relative dominance of their components; in a system transitioning to forest, the greenness and dryness/brightness components can mask peatland-specific hydrological degradation unless the analysis is explicitly habitat-stratified^68^. RSEI is also sensitive to short-term reflectance fluctuations and acquisition timing, which can bias long-term trends unless seasonality is carefully controlled^70^.

Although Tasseled Cap Wetness (TCW) is a physically interpretable optical proxy of canopy or soil moisture, it is insufficient for peatland hydrological assessment because optical wetness is strongly modulated by vegetation structure and phenology and suffers from cloud-driven data gaps, while it does not directly exploit water’s dielectric response. By contrast, SAR provides all-weather, day-night observations and is intrinsically sensitive to near-surface moisture, inundation and roughness via changes in radar backscatter, key controls of peatland hydrology, including snow or ice periods and seasonal flooding^78^. This is increasingly relevant in Poland, where warming is linked to lowered peatland water tables and higher drying risk, so “greening” can decouple from hydrological integrity^79^. Recent studies show Sentinel-1 backscatter supports monitoring water-table dynamics and wetness anomalies, and that fusing SAR with optical data improves inference relative to optical-only approaches. SAR variables are also among the most sensitive predictors of groundwater-level conditions in Polish peatlands^80^, and synthesis work likewise emphasizes hydrology-focused monitoring using SAR-based soil-moisture and drought indicators rather than optical proxies alone^81^. Accordingly, our Landsat/TCW results should be treated as a surface optical wetness proxy, and future monitoring of Bagna Izbickie should explicitly integrate SAR-derived wetness and roughness (e.g., Sentinel-1) alongside Landsat indicators to better constrain hydrological condition and its implications for carbon-sink functioning.

Future work can build on these insights by shifting emphasis from generic eco-environment indices toward peatland integrity metrics that directly target hydrology and open-bog structure. Two directions are particularly promising. The first is a class-explicit framework combining land-cover transitions (open bog to woody or forest states) with moisture-oriented optical proxies (e.g., TC Wetness, NDWI, NDMI-type indices), which would more directly link habitat change to surface wetness dynamics^11,48^. The second is multi-sensor integration with SAR, which has demonstrated capacity to improve inference on peatland wetness and water-table depth relative to optical-only approaches^64,82,83^. Such developments would strengthen causal attribution (climate pressure versus management effects) and reduce the risk of misinterpreting greening as peatland recovery.

## Conclusion

Using a multi-decadal Landsat archive, we documented a pronounced, directional shift in the Bagna Izbickie reserve from an open bog mosaic toward a forest‐dominated landscape between 1984 and 2024, with bog extent shrinking to a small fraction of its initial area while forest cover expanded. Placing these land-cover trajectories in a hydroclimatic context derived from TerraClimate supports the interpretation that progressive summer moisture limitation and surface warming have contributed to reduced resilience of remaining bog patches and facilitated woody encroachment. A key methodological outcome of this study is that composite remote sensed eco-environment indices such as the Remote Sensing Ecological Index (RSEI) can diverge from habitat-integrity trajectories in peatland landscapes undergoing peatland-to-forest transition. Because RSEI integrates greenness, wetness, dryness and heat through a PCA framework, it may increase at the landscape scale as canopy cover expands, even when open bog habitats contract and wetness signals in bog pixels do not show sustained improvement. This highlights an urgent need to develop and adopt wetland- and peatland-specific condition indicators that explicitly track peatland integrity (i.e., persistent near-surface wetness, suppression of woody encroachment, and maintenance of peat-forming vegetation) rather than relying on generic indices optimized for broad terrestrial vegetation dynamics. Such tailored indicators are essential for robust long-term monitoring and for evaluating management actions in a changing climate, particularly given the outsized role of peatlands in climate regulation and the large emissions associated with peatland drainage and degradation.

## Supplementary Information

Below is the link to the electronic supplementary material.

Supplementary Material 1

## Data Availability

The datasets generated during and analysed during the current study are available from the corresponding author on reasonable request.

## References

[1] Gorham, E. Northern peatlands: Role in the carbon cycle and probable responses to climatic warming. *Ecol. Appl.* **1**, 182–195 (1991). 27755660 10.2307/1941811

[2] Dunn, C., Freeman, C. & Peatlands Our greatest source of carbon credits? *Carbon Manag.* **2**, 289–301. 10.4155/cmt.11.23 (2011).

[3] Taillardat, P., Thompson, B. S., Garneau, M., Trottier, K. & Friess, D. A. Climate change mitigation potential of wetlands and the cost-effectiveness of their restoration. *Interface Focus*. **10**, 5. 10.1098/rsfs.2019.0129 (2020). 10.1098/rsfs.2019.0129 PMC7435041 32832065

[4] Winter, T. C. The vulnerability of wetlands to climate change: A hydrologic landscape perspective. *J. Am. Water Resour. Assoc.* **36**, 305–311. 10.1111/j.1752-1688.2000.tb04269.x (2000).

[5] Dai, A. Increasing drought under global warming in observations and models. *Nat. Clim. Change*. **3**, 52–58. 10.1038/nclimate1633 (2013).

[6] Stirling, E., Fitzpatrick, R. W. & Mosley, L. M. Drought effects on wet soils in inland wetlands and peatlands. *Earth Sci. Rev.* **210**10.1016/j.earscirev.2020.103387 (2020).

[7] Lawley, V., Lewis, M., Clarke, K. & Ostendorf, B. Site-based and remote sensing methods for monitoring indicators of vegetation condition: An Australian review. *Ecol. Ind.* **60**, 1273–1283. 10.1016/j.ecolind.2015.03.021 (2016).

[8] Dronova, I. Object-based image analysis in wetland research: A review. *Remote Sens.* **7**, 6380–6413. 10.3390/rs70506380 (2015).

[9] Klemas, V. V. Coastal and environmental remote sensing from unmanned aerial vehicles: An overview. *J. Coastal Res.* **31**, 1260–1267. 10.2112/JCOASTRES-D-15-00005.1 (2015).

[10] Guo, M., Li, J., Sheng, C., Xu, J. & Wu, L. A review of wetland remote sensing. *Sens. (Switzerland)*. **17**, 4. 10.3390/s17040777 (2017). 10.3390/s17040777 PMC5422050 28379174

[11] Czapiewski, S. & Szumińska, D. An overview of remote sensing data applications in peatland research based on works from the period 2010–2021. *Land* **11**, 1. 10.3390/land11010024 (2022).36211983

[12] Mahdavi, S. et al. Remote sensing for wetland classification: a comprehensive review. *GIScience Remote Sens.* **55**, 623–658. 10.1080/15481603.2017.1419602 (2018).

[13] Gallant, A. L. The challenges of remote monitoring of wetlands. *Remote Sens.* **7**, 10938–10950. 10.3390/rs70810938 (2015).

[14] Xu, H. A remote sensing urban ecological index and its application. *Acta Ecol. Sin.* **33** (24), 7853–7862. 10.5846/stxb201208301223 (2013).

[15] Jasnowski, M. *Peatlands of the Słupsk Voivodeship: Status, resources, importance, management principles, protection* (Agricultural University of Szczecin, 1990).

[16] Wojciechowski, A. Holocene deposits and molluscan assemblages in Lake Łebsko, Gardno-Łeba Coastal Plain. In K. Rotnicki (Ed.), *Polish Coast: Past, Present and Future. Journal of Coastal Research*, Special Issue 22, 236–243 (1995).

[17] Staszak-Piekarska, A. & Rzodkiewicz, M. Reconstruction of palaeoecological changes in Lake Łebsko on the basis of diatom analysis (the southern Baltic coast, Poland). *Landf. Anal.* **29**, 81–90. 10.12657/landfana.029.010 (2015).

[18] Pawlaczyk, P. *Conservation of the Baltic Raised Bogs in Pomerania, Poland: The first Polish LIFE-Nature project* (Naturalists’ Club Publishing House, 2007).

[19] Ilnicki, P. & Peatlands and peat. Poznań: Publishing House of the Agricultural University of Poznań (2002).

[20] Polish Geological Institute. *Geological Map of Poland 1:500,000 [Online: pgi.gov.pl]* (Polish Geological Institute, 2022).

[21] Chlost, I. & Sikora, M. The impact of anthropogenic pressure on the change of water relations in Gardno-Łeba Lowland. *Quaestiones Geographicae*. **34**, 17–31. 10.1515/quageo-2015-0030 (2015).

[22] Pawlaczyk, P., Herbichowa, M. & Stańsko, R. *Conservation of Baltic Bogs: A guide for practitioners, theorists, and officials* (Naturalists’ Club Publishing House, 2005).

[23] Chief Inspectorate of Environmental Protection. *Monitoring of species and natural habitats with particular emphasis on Natura 2000 areas* (Chief Inspectorate of Environmental Protection, 2018).

[24] Roy, D. P. et al. Landsat-8: Science and product vision for terrestrial global change research. *Remote Sens. Environ.* **145**, 154–172. 10.1016/j.rse.2014.02.001 (2014).

[25] U. S. G. S. Landsat Collection 2 Level-2 Science Products Product Guide/Documentation. *U S Geol. Surv. Fact. Sheet 2021–3055*. 10.3133/fs20213055 (2021).

[26] Roy, D. P. et al. Landsat-8: Science and product vision for terrestrial global change research. *Remote Sens. Environ.* **185**, 271–292 (2016).

[27] Wulder, M. A. et al. The global Landsat archive: Status, consolidation, and direction. *Remote Sens. Environ.* **185**, 271–283 (2016).

[28] Zhu, Z. & Woodcock, C. E. Continuous change detection and classification of land cover using all available Landsat data. *Remote Sens. Environ.* **144**, 152–171 (2014).

[29] Zhu, Z. & Woodcock, C. E. Object-based cloud and cloud shadow detection in Landsat imagery. *Remote Sens. Environ.* **118**, 83–94. 10.1016/j.rse.2011.10.028 (2012).

[30] Foga, S. et al. Cloud detection algorithm comparison and validation for operational Landsat data products. *Remote Sens. Environ.* **194**, 379–390. 10.1016/j.rse.2017.03.026 (2017).

[31] Huntington, J. L. et al. Climate Engine: Cloud computing for climate and remote sensing data analysis. *PLOS ONE*. **12**, e0184471 (2017).28886127

[32] Abatzoglou, J. T., Dobrowski, S. Z., Parks, S. A. & Hegewisch, K. C. TerraClimate, a high-resolution global dataset of monthly climate and climatic water balance from 1958–2015. *Sci. Data*. **5**, 170191. 10.1038/sdata.2017.191 (2018). 29313841 10.1038/sdata.2017.191 PMC5759372

[33] Wilks, D. S. *Statistical Methods in the Atmospheric Sciences* 3rd edn (Academic, 2011).

[34] Gorelick, N. et al. Google Earth Engine: Planetary-scale geospatial analysis for everyone. *Remote Sens. Environ.* **202**, 18–27. 10.1016/j.rse.2017.06.031 (2017).

[35] Wulder, M. A. et al. Current status of Landsat program, science, and applications. *Remote Sens. Environ.* **225**, 127–147 (2019).

[36] Rouse, J. W., Haas, R. H., Schell, J. A. & Deering, D. W. Monitoring vegetation systems in the Great Plains with ERTS. *Proc. Third Earth Resour. Technol. Satellite-1 Symp.* **1**, 309–317 (1974).

[37] Tucker, C. J. Red and photographic infrared linear combinations for monitoring vegetation. *Remote Sens. Environ.* **8**, 127–150. 10.1016/0034-4257(79)90013-0 (1979).

[38] Gao, B. C. NDWI — A normalized difference water index for remote sensing of vegetation liquid water from space. *Remote Sens. Environ.* **58**, 257–266. 10.1016/S0034-4257(96)00067-3 (1996).

[39] Key, C. H. & Benson, N. C. Landscape assessment: remote sensing of severity, the Normalized Burn Ratio. FIREMON: Fire Effects Monitoring and Inventory System (USDA Forest Service, RMRS-GTR-164-CD). (2006).

[40] Xu, H. Modification of normalized difference water index (NDWI) to enhance open water features in remotely sensed imagery. *Int. J. Remote Sens.* **27**, 3025–3033. 10.1080/01431160600589179 (2006).

[41] White, J. C. et al. Pixel-based image compositing for large-area dense time series applications and science. *Remote Sens. Environ.* **141**, 1–14 (2014).

[42] Breiman, L. Random forests. *Mach. Learn.* **45**, 5–32. 10.1023/A:1010933404324 (2001).

[43] Belgiu, M. & Drăguţ, L. Random forest in remote sensing: A review of applications and future directions. *ISPRS J. Photogrammetry Remote Sens.* **114**, 24–31. 10.1016/j.isprsjprs.2016.01.011 (2016).

[44] Congalton, R. G. & Green, K. *Assessing the Accuracy of Remotely Sensed Data: Principles and Practices* 2nd edn (CRC, 2009).

[45] Boriah, S., Kumar, V., Steinbach, M., Potter, C. & Klooster, S. Land cover change detection: A case study. Proceedings of the 14th ACM SIGKDD International Conference on Knowledge Discovery and Data Mining (KDD). (2008).

[46] Hu, X. & Xu, H. A. New Remote Sensing Index for Assessing the Spatial Heterogeneity in Urban Ecological Quality: A Case from Fuzhou City, China. *Ecol. Ind.* **89**, 11–21. 10.1016/j.ecolind.2018.02.006 (2018).

[47] Hao, H. et al. Ecological Index Approach for Restoration Assessment of Rare‐Earth Elements Mining. *Comput. Intell. Neurosci.* **2022** (1), 5335419. 10.1155/2022/5335419 (2022). 35875751 10.1155/2022/5335419 PMC9303088

[48] Crist, E. P. & Cicone, R. C. A physically-based transformation of Thematic Mapper data—The TM Tasseled Cap. *IEEE Trans. Geosci. Remote Sens.* **22**, 256–263. 10.1109/TGRS.1984.350619 (1984).

[49] Baig, M. H. A., Zhang, L., Shuai, T. & Tong, Q. Derivation of a tasseled cap transformation based on Landsat 8 at-satellite reflectance. *Remote Sens. Lett.* **5**, 423–431. 10.1080/2150704X.2014.915434 (2014).

[50] Weng, Q. Thermal infrared remote sensing for urban climate and environmental studies: Methods, applications, and trends. *ISPRS J. Photogrammetry Remote Sens.* **59**, 145–161 (2004).

[51] Li, Z. L. et al. Satellite-derived land surface temperature: Current status and perspectives. *Remote Sens. Environ.* **131**, 14–37. 10.1016/j.rse.2012.12.008 (2013).

[52] Jiang, H., He, G., Long, T., Liu, S. & Ni, Y. A new spectral index for bare soil mapping using Landsat imagery. *Remote Sens. Lett.* **10**, 365–374 (2019).

[53] Jin, W. et al. Continuous remote sensing ecological index (CRSEI): A novel approach for multitemporal monitoring of eco-environmental changes on large scale. *Ecol. Ind.* **154**, 110739. 10.1016/j.ecolind.2023.110739 (2023).

[54] Xu, E., Zhang, G., Wang, H., Yang, M., Tian, H., Zhao, M., … Wei, D. Monitoring and Assessing Ecological Environmental Quality in Qianping Reservoir, Central China: A Remote Sensing Ecological Index (RSEI) Approach. Forests16, 831. 10.3390/f16050831 (2025).

[55] Theil, H. A rank-invariant method of linear and polynomial regression analysis. *Indagationes Math.* **12** (85), 173 (1950).

[56] Sen, P. K. Estimates of the regression coefficient based on Kendall’s tau. *J. Am. Stat. Assoc.* **63** (324), 1379–1389 (1968).

[57] Mann, H. B. Nonparametric tests against trend. *Econometrica: J. econometric society*, 245–259. (1945).

[58] Hamed, K. H. & Rao, A. R. A modified Mann-Kendall trend test for autocorrelated data. *J. Hydrol.* **204** (1–4), 182–196. 10.1016/S0022-1694(97)00125-X (1998).

[59] Yue, S. & Wang, C. The Mann-Kendall test modified by effective sample size to detect trend in serially correlated hydrological series. *Water Resour. Manage*. **18** (3), 201–218. 10.1023/B:WARM.0000043140.61082.60 (2004).

[60] Nowakowska, J., Gazda, A., Tomski, A. & Szwagrzyk, J. Drainage ditches enhance forest succession in a raised bog but do not affect the spatial pattern of tree encroachment. *PLOS ONE*. **16**, e0247760. 10.1371/journal.pone.0247760 (2021). 33735308 10.1371/journal.pone.0247760 PMC7971578

[61] Dyderski, M. K., Gdula, A. K. & Jagodziński, A. M. Encroachment of woody species on a drained transitional peat bog in ‘Mszar Bogdaniec’ nature reserve (Western Poland). *Folia Forestalia Pol. Ser. – Forestry*. **57**, 161–173 (2015).

[62] Demarquet, Q., Rapinel, S., Dufour, S. & Hubert-Moy, L. Long-Term Wetland Monitoring Using the Landsat Archive: A Review. *Remote Sens.* **15**, 820. 10.3390/rs15030820 (2023).

[63] Šimanauskienė, R. et al. Peatland degradation: The relationship between raised bog hydrology and normalized difference vegetation index. *Ecohydrology* **12** (8), e2159. 10.1002/eco.2159 (2019).

[64] Räsänen, A., Tolvanen, A. & Kareksela, S. Monitoring peatland water table depth with optical and radar satellite imagery. *Int. J. Appl. Earth Obs. Geoinf.* **112**, 102866. 10.1016/j.jag.2022.102866 (2022).

[65] Olofsson, P., Foody, G. M., Stehman, S. V. & Woodcock, C. E. Making better use of accuracy data in land change studies: Estimating accuracy and area and quantifying uncertainty using stratified estimation. *Remote Sens. Environ.* **129**, 122–131. 10.1016/j.rse.2012.10.031 (2013).

[66] Olofsson, P. et al. Good practices for estimating area and assessing accuracy of land change. *Remote Sens. Environ.* **148**, 42–57. 10.1016/j.rse.2014.02.015 (2014).

[67] Intergovernmental Panel on Climate Change. *Climate change 2022: Impacts, adaptation and vulnerability* (Cambridge University Press, 2022).

[68] Wang, Z., Chen, T., Zhu, D., Jia, K. & Plaza, A. RSEIFE: A new remote sensing ecological index for simulating the land surface eco-environment. *J. Environ. Manage.* **326**, 116851. 10.1016/j.jenvman.2022.116851 (2023). 36442350 10.1016/j.jenvman.2022.116851

[69] Gong, X., Li, T., Wang, R., Hu, S. & Yuan, S. Beyond the remote sensing ecological index: A comprehensive ecological quality evaluation using a Deep-learning-based Remote Sensing Ecological Index. *Remote Sens.* **17**, 558. 10.3390/rs17030558 (2025).

[70] Sun, C. et al. Ecological quality assessment and monitoring using a time-series remote sensing-based ecological index(ts-RSEI). *GIScience Remote Sens.* **59**, 1793–1816. 10.1080/15481603.2022.2138010 (2022).

[71] Minasny, B., Adetsu, D. V., Aitkenhead, M., Artz, R. R., Baggaley, N., Barthelmes,A., … Zak, D. Mapping and monitoring peatland conditions from global to field scale.*Biogeochemistry*, 167(4), 383–425. 10.1007/s10533-023-01084-1 (2024).

[72] Dąbrowska-Zielińska, K. et al. Biophysical parameters assessed from microwave and optical data. *Int. J. Electron. Telecommunications*. **58** (2). 10.2478/v10177-012-0013-7 (2012).

[73] Toca, L. et al. Potential for peatland water table depth monitoring using Sentinel-1 SAR backscatter:case study of Forsinard Flows, Scotland, UK. *Remote Sens.* **15** (7), 1900. 10.3390/rs15071900 (2023).

[74] Pang, Y., Huang, Y., Zhou, Y., Xu, J. & Wu, Y. Identifying spectral features of characteristics of Sphagnum to assess the remote sensing potential of peatlands: A case study in China. *Mires Peat*. **26**, 25. 10.19189/MaP.2019.OMB.StA.1834 (2020).

[75] Lees, K. J. et al. Using spectral indices to estimate water content and GPP in Sphagnum moss and other peatland vegetation. *IEEE Trans. Geosci. Remote Sens.* **58**, 4547–4557. 10.1109/TGRS.2019.2961479 (2020).

[76] Dabrowska-Zielinska, K., Bartold, M. & Gurdak, R. POLWET—System for new space-based products for wetlands under RAMSAR Convention. *Geoinf. Issues*. **8**, 25–35. 10.34867/gi.2016.3 (2016).

[77] General Directorate for Environmental Protection (GDOŚ). (Geoserwis; WMS/WFS services). *Portal Gov. pl* (2026).

[78] Dabrowska-Zielinska, K., Budzynska, M., Tomaszewska, M., Bartold, M. & Gatkowska, M. The study of multifrequency microwave satellite images for vegetation biomass and humidity of the area under Ramsar convention. In 2015 IEEE International Geoscience and Remote Sensing Symposium (IGARSS) (pp. 5198–5200). IEEE. (2015), July.

[79] Pleskot, K., Apolinarska, K., Cwynar, L. C., Kotrys, B. & Lamentowicz, M. The late-Holocene relationship between peatland water table depth and summer temperature in northern Poland. *Palaeogeogr., Palaeoclimatol. Palaeoecol.* **586**, 110758. 10.1016/j.palaeo.2021.110758 (2022).

[80] Stachowicz, M. et al. Estimating mean groundwater levels in peatlands using a Bayesian belief network approach with remote sensing data. *Przegląd Naukowy Inżynieria i Kształtowanie Środowiska*. 33. 10.22630/srees.9939 (2024).

[81] de Waard, F., Connolly, J., Barthelmes, A., Joosten, H. & van der Linden, S. Remote sensing of peatland degradation in temperate and boreal climate zones–A review of the potentials, gaps, and challenges. *Ecol. Ind.* **166**, 112437. 10.1016/j.ecolind.2024.112437 (2024).

[82] Bartold, M. et al. Mapping management intensity types in grasslands with synergistic use of Sentinel–1 and Sentinel–2 satellite images. *Sci. Rep.* **14**, 32066. 10.1038/s41598-024-83699-4 (2024). 39738429 10.1038/s41598-024-83699-4 PMC11685408

[83] Ghezelayagh, P. et al. Developing a remote-sensing-based indicator for peat soil vertical displacement: A case study in the Biebrza Valley, Poland. *Ecol. Ind.* **166**, 112305. 10.1016/j.ecolind.2024.112305 (2024).

